# Supplementary material for: Phylogenetic Studies and Metabolite Analysis of Sticta Species from Colombia and Chile by Ultra-High Performance Liquid Chromatography-High Resolution-Q-Orbitrap-Mass Spectrometry
Source: Metabolites. 2022 Feb 8;12(2):156. doi: 10.3390/metabo12020156 (PMC8875025; doi:10.3390/metabo12020156)
Supplement: Supplementary file 1 [file metabolites-12-00156-s001.zip › metabolites-1580922-supplementary.pdf]

Supplementary Material

Phylogenetic studies and Metabolite Analysis of *Sticta* Species from Colombia and Chile by Ultra-High Performance Liquid Chromatography-High Resolution-Q-Orbitrap-Mass Spectrometry

Laura Albornoz[1¥], Alfredo Torres-Benítez[2¥], Miguel Moreno-Palacios[3], Mario J. Simirgiotis[2], Saúl A. Montoya S.[4], Beatriz Sepulveda[5], Elena Stashenko[6], Olimpo García-Beltrán[7,8]* and Carlos Areche[1]*

[1] Departamento de Química, Facultad de Ciencias, Universidad de Chile, Las Palmeras 3425, Nuñoa, Santiago 7800024. E mail: albornoznocachomail.c@hotmail.com (L.A), areche@uchile.cl (C.A).

[2] Instituto de Farmacia, Facultad de Ciencias, Universidad Austral de Chile, Campus Isla Teja 5090000, Valdivia, Chile. E mail: alfredo.torres@unibague.edu.co (A.T.B), mario.simirgiotis@uach.cl (M.J.S)

[3] Laboratorio de Biología Evolutiva de Vertebrados, Departamento de Ciencias Biológicas, Universidad de Los Andes, Bogotá, Colombia. E mail: mc.morenop@uniandes.edu.co (M.M.-P.)

[4] Laboratorio de toxicología, seccional Tolima, Instituto Nacional de Medicina Legal y Ciencias Forenses. Ibagué, Colombia. E mail: samontoya@medicinalegal.gov.co (S.A.M.S)

[5] Departamento de Ciencias Químicas, Universidad Andres Bello, Campus Viña del Mar, Quillota 980, Viña del Mar, Chile. Email: bsepulveda@uc.cl (B.S.)

[6] Research Center of Excellence CENIVAM, CIBIMOL, Universidad Industrial de Santander, Building 45, UIS, Carrera 27, Calle 9, Bucaramanga 680002, Colombia; elena@tucan.uis.edu.co

[7] Universidad Bernardo O'Higgins, Centro Integrativo de Biología y Química Aplicada (CIBQA), General Gana 1702, Santiago, Chile, 8370854.

[8] Facultad de Ciencias Naturales y Matemáticas, Universidad de Ibagué, Carrera 22 Calle 67, Ibagué 730002, Colombia

[¥] These two authors share first authorship

* Correspondence: jose.garcia@unibague.edu.co (O.G.-B.); areche@uchile.cl (C.A). Tel.: +57-8276-0010 (O.G.-B.); +56 988377059 (C.A.

Supplementary table S1. Description of character and character states used in the present study of *Sticta*

Character 1	Primary photobiont	
Character status	Green alga	1
	Cyanobacteria	2

Character 2	Peduncle	
Character status	Absent	0
	Indistinct	1
	Absent to indistinct	2

Character 3	Thallus	
Character status	Irregular, moderately branched, 3-5 branched, anisotomous to pleurotomous type.	1
	Irregular, moderately branched, 3-5 branching, anisotomic to polytomic type	2
	Irregular, highly branched, 6-10 branching, polytomous to pleurotomous type	3
	Irregular, much branched, 6-10 branched, pleurotomical to dichotomous type	4
	Irregular to suborbicular, moderately branched, 3-5 branching, anisotomic to pleurotomic type	5
	Irregular to suborbicular, highly branched, 6-10 branched, anisotomous to pleurotomous type	6
	Orbicular, sparsely branched, 0-2 branched, polytomous type	7
	Orbicular to suborbicular, sparsely branched, 0-2 branched, polytomous to anisotomous type	8
	Palmate, moderately branched, 3-5 branched, anisotomic to polytomic type	9

Character 4	Lobes	
Character status	Lacinate to ligulate, ascending to subpendulous, interspaced to adjacent, revolute, apices rounded to obtuse, margins entire, internodes of lobes papillose-fragile	1
	Lacinate to ligulate, adnate, imbricate, flat to involute, apices rounded, margins entire to sinuous, internodes of subcoriaceous-fragile lobes	2
	Lacinate to ligulate, horizontal to subpendulous, imbricate, wavy, rounded apices, margins entire, internodes of coriaceous-resistant lobes	3
	Ligulate to flabellate, horizontal to slightly ascending, imbricate, wavy to involucrate, apices rounded to truncate, margins entire to sinuous, internode of leathery-fragile lobes	4
	Ligulate to flabellate, adnate to slightly ascending, imbricate, involucrate to slightly caniculate, apices rounded, margins entire to crenulate, internode of leathery lobes-resistant	5
	Ligulate to flabellate, adnate, adjacent to imbricate, flat to wavy, apices rounded, margins entire to crenulate, internode of leathery-fragile lobes	6
	Ligulate, subpendulous, adjacent to imbricate, flat, apices obtuse to acute, margins entire, internode of coriaceous-fragile lobes	7

	Flabellate to ligulate, adnate to horizontal, adjacent to imbricate, wavy, rounded apices, entire to sinuous margins, internodes of leathery-resistant lobes	8
	Flabellate, ascending to subpendulous, imbricate, involucrate to caniculate, apices rounded, margins entire to crenulate, internodes of lobes papyrose-fragile	9
	Suborbicular, horizontal to slightly ascending, imbricate, involuting to undulate, apices rounded, margins entire, internodes of lobes coriaceous-fragile	10
	Suborbicular, horizontal to subpendicular, imbricate, wavy, rounded apices, margins entire, internode of lobes subcoriaceous to coriaceous-fragile	11

Character 5	Superior surface	
Character status	Smooth to laxly scrobiculate, yellow-green, glabrous surface, with or without sparse papillae, without pruina, without macules or with cream irregular macules	1
	Smooth to slightly scrobiculated, brownish green, surface pubescent to scabrous, without papillae, without pruina, with white irregular macules	2
	Smooth - scrobiculated to slightly rugose towards the center, greenish-gray, glabrous surface, without papillae, without pruina, with irregular white macules	3
	Smooth to ribbed, greenish brown, scabrous surface, without papillae, without pruina, with cream irregular macules	4
	Smooth to centrally rough, bluish-green, glabrous surface, no papillae, no pruina, with cream irregular macules	5
	Rough to slightly scrobiculated, greenish brown, glabrous surface, without papillae to sparse, without pruina, with cream irregular macules	6
	Rough to smooth, brownish gray, surface glabrous, no papillae, no pruina, with cream irregular macules	7
	Foveolate to centrally rugose, dark green to brownish green, glabrous surface, with or without sparse papillae and in some cases papillotrichomes, without pruina, with cream irregular macules	8
	Scrobiculate to faveolate, yellow-green, glabrous surface, without papillae, without pruina, with irregular white maculae	9
	Scrobiculate to slightly rough, yellow-green, glabrous surface, without papillae, without pruina, with irregular cream macules	10
	Scrobiculate to rough or wavy, spermy green, arachnoid to spongy surface, without papillae, without pruina, with cream irregular macules	11

Character 6	Cilia	
Character status	Absent	0
	Scarce	1
	Abundant to absent	2
	Abundant to scarce	3
	Abundant	4

Character 7	Apothecia

Character status	Sparse	1
	Scanty to abundant, submarginal, sparse, subpedicellate, slight lower basal invagination, disc reddish-brown-brilliant brown, margin entire to slightly tomentose pale yellowish-brown pubescent	2
	Abundant, laminar, sparse, pedicellate, pronounced basal invagination, disc orange to reddish-reddish-bright reddish-brown, margin cream to beige hirsute	3
	Abundant, laminar, sparse, pedicellate, pronounced lower basal invagination, orange to reddish-orange-opaque disc, white to cream tomentose to hirsute margin	4
	Abundant, laminar, sparse, subpedicellate, pronounced lower basal invagination, disc reddish-brown to dark brown-bright, margin hirsute-tomentose yellowish brown	5
	Abundant, laminar, aggregate, pedicellate, pronounced lower basal invagination, disc reddish-brown to dull brown, margin hirsute to entire brown and creamy white	6
	Abundant, laminar, sessile to subpedicellate, slight lower basal invagination, disc orange to reddish-brown-opaque to shiny, margin entire crenulate to creamy white tomentose	7
	Abundant, submarginal, clustered to sparse, subpedicellate, pronounced lower basal invagination, orange to orange brown-opaque disc, crenulate to slightly hirsute pale ochre to dark brown margin	8
	Abundant, submarginal, aggregate, subpedicellate, pronounced lower invagination, disc orange-brown to reddish-brown-opaque, upper margin crenulate and lower margin hirsute creamy white to beige	9

Character 8	Vegetative propagules	
Character status	Absent	0
	Marginal, aggregates, pale blue-grayish color	1
	Laminates, aggregates, dark brownish-green color	2
	Marginals, dark brown aggregates	3

Character 9	Medulla	
Character status	Lax	1
	Compact	2
	Lax to compact	3

Character 10	Lower surface	
Character status	Smooth to veined cream yellow	1
	Smooth to wavy cream to light brown	2
	Smooth to wavy cream to dark brown	3
	Smooth to wavy white to grayish-brown	4
	Wavy cream-colored	5
	Wavy black	6

	Cream-white to beige-brown wavy	7
	Wavy cream-white to cream-white	8
	Wavy to light-brown ribbed	9
	Wavy to ribbed beige to dark brown	10

Character 11	Primary tomentum	
Character status	Sparse, thin, pubescent to fasciculate, rough, cream colored	1
	Sparse, thin, spongy to arachnoid, coarse, dark brown	2
	Sparse, thin, spongy, soft, golden-brown to dark brown	3
	Irregular, coarse, spongy to fasciculate, soft, grayish brown	4
	Irregular, coarse, spongy to fasciculate, soft, white to grayish white	5
	Dense, coarse, spongy, rough, gray-brown to dark gray-brown	6
	Dense, coarse, spongy, soft, blackish brown	7
	Dense, coarse, spongy, soft, cream colored	8
	Dense, coarse, spongy to fasciculate, soft, cream-white to grayish brown	9

Character 12	Secondary tomentum	
Character status	Absent	0
	Arachnoid, clear	1
	Pubescent, clear	2

Character 13	Rhizines	
Character status	Absent	0
	Few white to cream colored marginals	1
	Scanty marginal black-brown	2
	Sparse scattered white to cream	3
	Abundant central black to brown	4
	Abundant scattered white	5

Character 14	Cyphellae	
Character status	Sparse, scattered, rounded to angular, immersed to prominent, margin without tomentum, basement membrane pubescent to yellow pruinose	1
	Abundant, scattered, rounded to angular, immersed to prominent, margin with tomentum, white pubescent basement membrane	2
	Abundant, scattered, rounded to angular, erumpent to suprasessile, margin without tomentum, white pubescent basement membrane	3
	Abundant, scattered, rounded to angular, erumpent to sessile, margin without tomentum, cream pubescent basement membrane	4
	Abundant, scattered, rounded, immersed to erumpent, margin without tomentum, white pubescent basement membrane	5
	Abundant, scattered, rounded, immersed to erumpent, margin with tomentum, white pubescent basement membrane	6

	Abundant, scattered, rounded, immersed to prominent, margin without tomentum, white to cream or yellowish pubescent basement membrane	7
	Abundant, scattered, rounded, prominent to sessile, margin without tomentum, basement membrane pubescent white	8
	Abundant, scattered, rounded to irregular, immersed to prominent, margin without tomentum, basement membrane pubescent cream colored	9
	Abundant, scattered, rounded to irregular, immersed to erumpent, margin with tomentum, white to cream colored pubescent basement membrane	10
	Abundant, scattered, angular to rounded, prominent to suprasessile, margin without tomentum, basement membrane pubescent white	11

Character 15	Cephalodia	
Character status	Present	1
	Absent	0

Character 16	Pycnidia	
Character status	Present	1
	Absent	0

Supplementary table S2. Character state matrix of morphological traits used to recover phylogenetic relationships in species of *Sticta*

ESPECIE/CARACTER	1	2	3	4	5	6	7	8	9	10	11	12	13	14	15	16
Sticta leariloba	1	1	1	1	1	0	2	0	1	1	1	0	1	1	1	1
Sticta impressula	2	0	3	4	8	4	3	0	2	7	6	0	0	3	0	1
Sticta ocaniensis	1	0	4	7	9	1	6	0	2	9	2	0	2	5	1	1
Sticta cf. andina	2	0	5	8	6	2	8	0	2	6	7	1	4	8	0	1
Sticta cf. hypoglabra	2	0	5	2	7	0	1	1	2	2	4	1	0	9	0	1
Sticta cordillerana	1	0	1	3	A	3	5	0	2	A	3	0	3	4	1	1
Sticta cf. gyalocarpa	2	0	7	A	2	4	9	0	3	4	5	1	0	2	0	0
Sticta leucoblepharis	2	2	9	9	3	4	7	0	1	8	9	2	0	B	0	0
Sticta parahumboldtii	2	0	8	B	B	4	4	0	2	5	8	1	0	A	0	1
Sticta pseudosylvatica	2	0	2	5	4	0	1	2	2	3	9	2	0	6	0	1
Sticta luteocyphellata	2	0	6	6	5	0	1	3	2	3	9	1	5	7	0	1
